# Optimizing tuberculosis post-exposure follow-up among healthcare personnel using a risk-based approach

**DOI:** 10.1017/ice.2026.10403

**Published:** 2026-05

**Authors:** Ayesha Samreen, Melanie D. Swift, Laura E. Breeher, Debra K. Apenhorst, Jenna M. Rasmusson, April R. Loeffler, Jennifer A. Anderson, Aditya S. Shah

**Affiliations:** 1 Division of Public Health, Infectious Diseases and Occupational Medicine, Mayo Clinic, Rochester, MN, USA; 2 Department of Nursing, Mayo Clinic, Rochester, MN, USA; 3 Infection Prevention and Control, Mayo Clinic, Rochester, MN, USA; 4 Employee Occupational Health Services, Mayo Clinic, Rochester, MN, USA

## Abstract

Healthcare personnel (HCP) are at risk for occupational exposure to tuberculosis. Current guidelines for managing exposed HCP are broad and resource intensive. Based on review of our internal data, we propose a risk-based stratification approach to streamline exposure follow-up testing and optimize resource utilization.

## Background

Tuberculosis (TB) is a highly communicable disease, and transmission in healthcare settings is well documented. The annual incidence of TB among HCP is estimated at 67, 91, and 1180 cases per 100,000 people in countries with low, intermediate, and high incidence of TB, respectively.^1^ The risk in the United States is 2/100,000 persons.^2,3^ Healthcare personnel (HCP) exposed to TB not only have health risks for themselves but may inadvertently expose vulnerable patients if the HCP develops active disease. This necessitates close coordination between occupational health services (OHS) and infection prevention and control (IPAC), with formal policies for managing exposure follow ups (EFUs).

Guidelines from the Centers for Disease Control and Prevention (CDC) recommend assessing exposure and risk in consultation with local TB programs and public health partners, considering factors such as the index case, exposure characteristics, HCP susceptibility, and institutional policies.^4,5^ However, they do not define minimum exposure times for follow-up testing and often leave prioritization to local programs. Guidelines prioritize HCP present during high-risk procedures (bronchoscopy, sputum induction, other aerosol generating procedures, autopsy, surgery) and consider prior TB screening, exposure intensity, and drug resistance patterns.

Because public health guidance is non-prescriptive, hospitals often adopt conservative approaches, with high volume but low yield testing, increasing resource utilization and HCP anxiety. Our healthcare campus, classified as medium-risk for TB, has observed TB transmission only from AFB (Acid fast bacilli) smear-positive, symptomatic patients in the last decade. To optimize EFU, we describe a risk-based stratification approach reflecting the most common exposure scenarios, aiming to streamline follow-up while maintaining safety (Table 1).

**Table 1.** Risk-stratification criteria for healthcare personnel (HCP) tuberculosis exposure follow up testing

Risk group	Exposure risk factors (to be identified by IPAC) patient contact	Recommended threshold of unprotected^a^ exposure^b^ to unmasked source patient to trigger mandatory postexposure testing
1	Aerosol generating procedure (AGP) Laryngeal TB or Pulmonary cavitary TB or Lung abscess or AFB smear positive with cough or Any MDR^c^ TB Lab specimen contact or Manipulation of TB culture outside biosafety cabinet or Splash of infectious clinical specimen (eg, sputum) to mucous membrane or Microtoming frozen section TB specimens outside biosafety cabinet	Any duration
2	AFB smear positive without cough	15 min
3	AFB smear negative	4 h

a Unprotected means not using a respirator.

b Exposure proximity = in same room with patient.

c Susceptibility for Multi-Drug Resistant (MDR) TB can take time to result and if MDR TB is identified, then IPAC will reach out again.

## Methods

We reviewed internal data on TB exposure–related testing from 2012 through 2022 to assess occupational TB risk, identify source and exposure characteristics linked to transmission, and quantify resources used in EFU. During this time, there was no clear guidance on exposure duration or risk level to prompt HCP screening. In July 2023, OHS and IPAC developed a risk-based exposure approach endorsed by the Minnesota State Department of Health. This approach incorporates clinical presentations of TB, duration of unprotected exposure to an unmasked source, and other risk stratification elements to guide HCP postexposure testing (Table 1). Each TB EFU was reviewed after implementation to assess the appropriateness of testing.

OHS staff review prior TB test history, educate exposed HCP, and arrange appropriate baseline and postexposure testing as needed. Activities such as tracking test status, sending reminders, arranging testing for HCP who have left the organization since exposure, and interpreting test results continue until all employees complete their follow-up testing. OHS nurse time ranges from 20 minutes to 2 hours per employee, increasing substantially in the rare event of a TB test conversion. Two 18-month periods were analyzed pre- and postimplementation: January 2022–June 2023 and July 2023–December 2024. Differences between periods were assessed using two tailed Mann-Whitney U test [R v4.3.1].^8^

## Results

Preimplementation, 8 EFU events impacted 147 HCP (mean 18.4 ± 11.85, median 16 (IQR 8–30.5) HCP/event) with no TB conversions. Of these exposures, 5 involved patients with pulmonary TB only, and 3 with both pulmonary and extrapulmonary TB. During this period, although risk-based exposure duration thresholds were not applied, a review of cases shows that four would be under Risk Group 1); two under Risk Group 2 and two in Risk Group 3. This suggests that if risk-based thresholds had been applied, EFU testing might not have been needed for lower-risk exposures (eg, Risk Group 3, AFB smear-negative), while still capturing clinically significant cases in Risk Groups 1 and 2.

**Figure 1.**
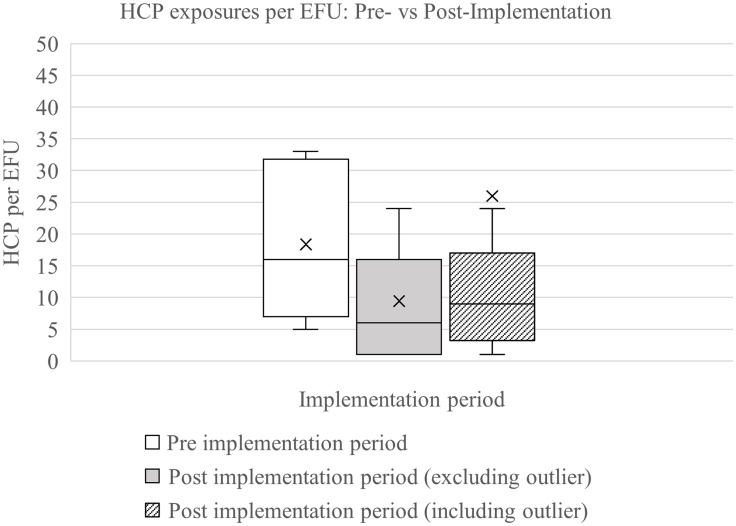
Distribution of healthcare personnel (HCP) exposures per exposure follow-up (EFU) event before and after implementation. Box-and-whisker plot showing the distribution of HCP exposures per EFU during the preimplementation period and postimplementation period (with and without inclusion of a single outlier). Boxes represent the interquartile range (IQR), horizontal lines within boxes indicate median values, and whiskers denote the minimum and maximum values within 1.5 × IQR. X markers indicate values beyond 1.5 × IQR.

Following implementation, 12 EFU events involved 104 HCP exposures (excluding an outlier event), to patients with pulmonary TB only (mean 8.7 ± 8.17; median 6 (IQR 1–14.5). This represents an overall reduction in the median number of HCP exposures per EFU from 16 (IQR, 8–30.5) in the preimplementation period to 6 (IQR, 1–14.5) postimplementation (*U* = 11.5; *p* = .005). (Mann–Whitney *U* = 11.5; *p* = .005). An “outlier” event in July 2024 was excluded from the primary analysis for reasons detailed in the Discussion, but included in a secondary analysis. When including this event in the analysis, the median was 6 (IQR, 1–20), and the difference remained statistically significant (*U* = 16; *p* = .007), as illustrated in Figure 1. These results indicate a significant reduction in HCP exposures associated with the intervention, even when including the outlier event.

Postimplementation, EFU events stratified by risk category included 6 EFU events with 31 HCP exposed in Risk Group 1, 2 EFU events in Risk Group 2 with 36 HCP exposed and 4 EFU events in Risk Group 3 with 37 HCP exposed. One HCP conversion occurred, in the Risk Group 1 outlier event.

## Discussion

Occupational exposures to TB in healthcare institutions require substantial resource utilization to conduct postexposure testing of HCP. Some testing is low yield as transmission is unlikely to occur from AFB smear-negative, asymptomatic patients. Beyond direct testing costs, additional burdens include staff time for communication and follow-up testing, and diversion of OHS resources from other clinical and programmatic activities.

We developed a risk- and time-based approach to define exposures that prompt EFU informed by internal stakeholder discussions, consultation with state public health authorities, and lessons learned from the COVID-19 pandemic^6,7^ (Table 1). Applying these criteria not only reduced the number of HCP involved in EFUs but also ensured that appropriate personnel were tested, while maintaining effective TB control (Figure 1).

During the postimplementation period, one event resulted in an unusually high number of exposed HCP due to delayed suspicion for TB. This “outlier” event involved a patient admitted for elective surgery whose follow-up chest computed tomography (CT) incidentally revealed an asymptomatic lung abscess prompting evaluation for pulmonary TB. Bronchoscopy specimens were AFB smear negative, but culture positive. The patient was hospitalized for 9 days without isolation prior to concern for TB, with potential exposure across multiple inpatient units. Due to the lung abscess, the exposure was classified as Risk Group 1, requiring testing of all HCP with any duration of unprotected exposure. Out of 208 exposed HCP, only one occupational conversion occurred, in a nurse who placed a nasogastric tube. The outlier event was excluded from the primary analysis since it included twice as many exposed HCP as all other exposures combined, potentially skewing the results. The protocol was designed to target common scenarios and not rare large-scale exposures. In this instance, our EFU testing process identified the TB conversion effectively. This case underscores the need for preparedness for rare, large-scale exposure events. However, even including the outlier event in our analysis, we observed a significant reduction in mean number of exposed HCP per event.

Thus, our findings support a focused, clinically effective EFU strategy that optimizes resource use and identifies appropriate staff for testing. This approach may serve as a framework for re-evaluating exposure guidelines for other respiratory pathogens in healthcare settings, although the small sample size limits generalizability. We note that no conversions occurred in Risk Group 3 (source patients who were AFB smear negative), indicating that surveillance of exposed individuals in this group is a conservative measure. Ongoing auditing, iterative review of data, and stakeholder engagement will guide future refinements to our approach.
